# The evolving role of DNA damage response in overcoming therapeutic resistance in ovarian cancer

**DOI:** 10.20517/cdr.2022.146

**Published:** 2023-06-14

**Authors:** Sara Bouberhan, Liron Bar-Peled, Yusuke Matoba, Varvara Mazina, Lauren Philp, Bo R. Rueda

**Affiliations:** ^1^Division of Hematology/Oncology, Department of Medicine, Massachusetts General Hospital, Boston, MA 02114, USA.; ^2^Department of Medicine, Harvard Medical School, Boston, MA 02115, USA.; ^3^Center for Cancer Research, Massachusetts General Hospital, Boston, MA 02114, USA.; ^4^Department of Obstetrics and Gynecology, Vincent Center for Reproductive Biology, Massachusetts General Hospital, Boston, MA 02114, USA.; ^5^Obstetrics, Gynecology, and Reproductive Biology, Harvard Medical School, Boston, MA 02115 USA.; ^6^Division of Gynecologic Oncology, Department of Obstetrics and Gynecology, Massachusetts General Hospital, Boston, MA 02114, USA.

**Keywords:** Ovarian cancer, platinum resistance, PARPi resistance, DDR

## Abstract

Epithelial ovarian cancer (EOC) is treated in the first-line setting with combined platinum and taxane chemotherapy, often followed by a maintenance poly (ADP-ribose) polymerase inhibitor (PARPi). Responses to first-line treatment are frequent. For many patients, however, responses are suboptimal or short-lived. Over the last several years, multiple new classes of agents targeting DNA damage response (DDR) mechanisms have advanced through clinical development. In this review, we explore the preclinical rationale for the use of ATR inhibitors, CHK1 inhibitors, and WEE1 inhibitors, emphasizing their application to chemotherapy-resistant and PARPi-resistant ovarian cancer. We also present an overview of the clinical development of the leading drugs in each of these classes, emphasizing the rationale for monotherapy and combination therapy approaches.

## OVARIAN CANCER AND PLATINUM-BASED CHEMOTHERAPY

Ovarian cancer is the deadliest gynecologic malignancy, estimated to account for 12,810 deaths in 2022^[1]^. Epithelial ovarian cancer (EOC) is treated in the first-line setting with combined platinum and taxane chemotherapy^[2]^. Over 80% of high-grade serous ovarian cancers (HGSOC), the most common ovarian cancer subtype, will exhibit an initial response to platinum-based chemotherapy^[3]^. Defects in homologous recombination repair (HRR) are present in about 50% of EOC, and the initial sensitivity of EOC to platinum-based therapy has been attributed to the high prevalence of homologous recombination deficiency (HRD) in this cancer type^[4]^. Only a minority (10%-15%) of HGSOC will not demonstrate a meaningful initial response to first-line platinum-based chemotherapy^[3]^. Regardless of the initial response to platinum-based chemotherapy, the majority of patients with EOC will go on to develop disease recurrence. Most recurrent EOC will eventually exhibit platinum resistance following treatment with one or more lines of chemotherapy^[2]^. In this regard, ovarian cancer exhibits both intrinsic and acquired resistance to platinum-based chemotherapy.

Multiple mechanisms of inherent and acquired resistance to platinum-based chemotherapy have been described. One of these is the alteration of volume-regulated anion channels (VRACs) which regulate the influx of platinum compounds into cells^[5,6]^. Additional mechanisms of platinum resistance include altered intracellular sequestration of platinum, changes to the tumor microenvironment, and altered recognition and repair of DNA damage^[4,5]^. Alterations in the DNA damage response (DDR) are being studied as a potential vulnerability that can be exploited for the treatment of EOC. It has been demonstrated that acquired alterations in key DDR genes following PARPi therapy, like reversion mutations in *BRCA, RAD51C*, and *RAD51D,* resulting in restoration of homologous recombination, are associated with the development of post-treatment resistance^[7]^. In this review, we will focus on the role of DDR in the current landscape of treatment for recurrent EOC.

## PARP INHIBITION IN OVARIAN CANCER

PARP inhibitors were the first drug class to exploit synthetic lethality for the treatment of ovarian cancer^[8]^. Synthetic lethality is the concept whereby an inactivating mutation in one gene (or inhibition of its protein product) is innocuous, but inactivating mutations in two genes (and/or inhibition of their protein products) results in cell death^[8]^. The proteins encoded by the *BRCA1* and *BRCA2* genes are critical for homologous recombination, a DNA damage repair pathway for double-strand break repair. The PARP1 protein is involved in single-strand break repair via base excision repair^[9]^. A PARPi is lethal only to cells with a predisposing defect in homologous recombination^[8]^. PARPi therapy has been most effective in patients with germline *BRCA1* or *BRCA2* mutations, whose tumors likely exhibit deficient homologous recombination. Maintenance therapy with the PARPi olaparib after first-line chemotherapy in patients with *BRCA-*mutated advanced ovarian cancer showed dramatic improvements in progression-free survival (PFS) compared to placebo^[10]^. Patients with germline *BRCA1* or *BRCA2* mutations account for about only 15% of all patients with ovarian cancer, but approximately 50% of epithelial ovarian cancers harbor defects in HRR^[11,12]^. PARP inhibitors have shown some activity in patients who do not carry *BRCA* mutations but whose tumors exhibit HRD based on the result of a somatic profiling assay (ex. Myriad MyChoice CDx HRD)^[10,13,14]^. On April 29, 2020, the FDA approved niraparib as first-line maintenance for all advanced EOC following first-line platinum-based chemotherapy, regardless of *BRCA* status or HRD status. The EMA made a similar approval on October 29, 2020.

The majority of patients treated with PARP inhibitors in the first-line setting will go on to experience disease recurrence^[10,14]^. Multiple mechanisms of PARPi resistance have been described. These include reversion mutations in *BRCA1* or *BRCA2*, which restore HRD^[15]^, downregulation of non-homologous end-joining (NHEJ), loss of 53BP1^[16]^, and enhanced replication fork protection^[17,18]^. Cells that exhibit HRD, such as *BRCA* deficient cells, can also defer to a more error-prone polymerase theta-mediated end-joining (TMEJ, a.k.a. alt-NHEJ or microhomology-mediated end-joining, MMEJ) as a backup pathway to repair double-strand breaks. TMEJ-mediated repair involves PARP1, DNA ligase III, and DNA polymerase theta (Polθ)^[19]^.

Polθ is encoded by the gene *POLQ.* Polθ has become a therapeutic target of interest in cancers due to evidence of synthetic lethality when there is a loss of *POLQ* and dysregulation or loss of other DNA repair-related tumor suppressor genes that control double-strand break repair or HRR^[20]^. Some recent studies suggest that secondary mutations restoring BRCA1/2 function are caused by the activity of TMEJ with Polθ facilitation. Ceccaldi *et al*. provided *in vitro* evidence that HRR-deficient ovarian cancer cells were dependent on Polθ^[21]^. ART558, a small molecule inhibitor of Polθ, elicited DNA damage in *BRCA1-* and *BRCA2-* mutant tumor cells and was shown to enhance the effects of a PARPi in *in vitro* and *in vivo* models^[22]^. ART 558 is expected to move forward in Phase I trials. It has also been proposed that cancer stem cells (CSCs) might display an inherent or acquired resistance to PARPi. Using *in vitro* and *in vivo* preclinical models of ovarian cancer, it was shown that PARP inhibition primarily targeted the non-cancer stem cell populations. While the CSCs showed increased evidence of DNA damage in response to PARP inhibition, the CSCs were able to repair their damaged DNA more efficiently than their non cancer stem cell populations^[23]^. DMC1, a meiotic-specific recombinase, which has been shown to be expressed in cancers, was proposed as a possible mediator of this purported resistance. Interestingly, recent research shows that diverse mechanisms of resistance can emerge within a *BRCA1* mutant cell line, and multiple mechanisms of resistance may emerge within even a single clone. For example, a single clone has been shown to demonstrate restoration of RAD51 foci formation and decreased levels of PARylation, suggesting downregulation of PARP1^[17]^. Given the multiple described mechanisms of acquired resistance to targeted small molecule inhibitors, overcoming therapeutic resistance is an ongoing challenge to investigators and clinicians alike^[24,25]^. Other combination strategies designed to target the CSCs as well as the more differentiated tumors cells have been investigated in preclinical ovarian cancer models, including those targeting the metabolic pathway, aldehyde dehydrogenase activity, and long non-coding RNAs in combination with either a cytotoxic or another biologic agent^[26-28]^. Others have shown that metformin could reduce CSC populations and increase sensitivity to cisplatin^[29]^. The diversity in the CSC populations from patient to patient is likely to have some impact on their success in the clinic.

## TARGETING DDR PROTEINS IN THE PARPI-RESISTANT OR PLATINUM-RESISTANT SETTING

The current treatment paradigm for PARPi-resistant and platinum-resistant HGSOC does not address specific mechanisms of resistance. Instead, treatment typically shifts away from therapies dependent on deficient HRR^[30]^. To this effect, PARP inhibitors have shown very poor efficacy in the heavily pretreated setting^[31]^. These findings suggest the need for an alternative approach to treatment for EOC that is resistant to both platinum-based chemotherapy and PARP inhibitors.

It is well understood that the cancer genome is less stable than that of healthy cells^[32]^, and tumor cells demonstrate high rates of DNA replication and division. HGSOC has a high incidence of inactivating *TP53* mutations^[33]^. *TP53* encodes p53, a critical tumor suppressor that protects cells from proceeding through the cell cycle in the setting of DNA damage^[34]^. The cooccurrence of inactivating *TP53* mutations and mutations in *BRCA1* and *BRCA2* has been suggested to be a critical element in the development of *BRCA* null tumors^[35,36]^. As such, PARPi resistance almost always occurs in the setting of deficient p53. In the setting of DNA damage, p53-deficient cells will be preferentially dependent on alternative cell cycle checkpoints. This selective dependence is being explored as a potential target for EOC treatment^[37,38]^.

While many mechanisms are likely at play, cancer cells ultimately attempt to replicate damaged DNA, leading to “replicative stress”. Genomic instability, resulting from an accumulation of DNA damage, is a hallmark of many cancers, including ovarian cancer. Accumulation of DNA damage can result in replication stress. ATM and ATR are kinases that the cells rely on to alleviate replication stress. Specifically, ATM/ATR signaling arrests the cell cycle via phosphorylation of downstream kinases such as CHK2 and CHK1, respectively. If ATM/ATR fails, the replication fork becomes unstable and can collapse^[39,40]^. To this end, these proteins critical to DDR (ATR, ATM, CHK1, CHK2) are being studied as potential therapeutic targets. This strategy relies on the hypothesis that inhibition of various DDR pathways may confer a therapeutic effect in heavily pretreated EOC that is resistant to either platinum-based chemotherapy, PARP inhibitors, or both. Inhibitors of ATR, ATM, CHK1, CHK2, and WEE1 are in clinical trials. Here we discuss inhibitors of ATR, CHK1, and WEE1 in more detail, as they have progressed the furthest in clinical development.

## ATR

**Figure 1:**
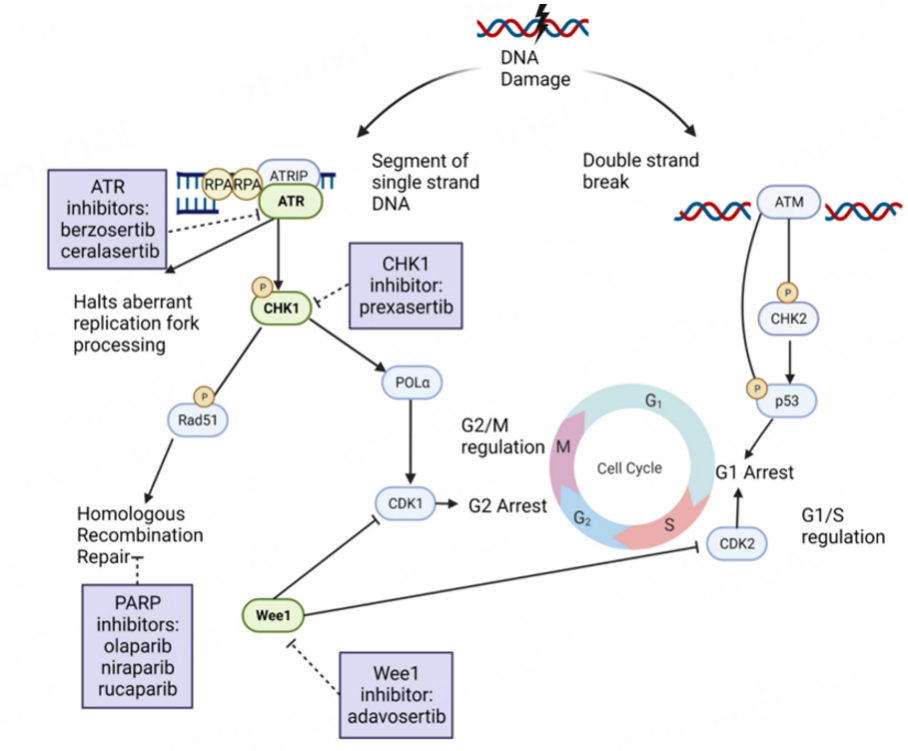
A schematic representation of the DNA damage response and its interaction with cell cycle regulation. RPA binds to sites of ssDNA and co-localizes with ATR and its binding partner ATRIP. CHK1 is the effector of ATR and regulates cell cycle progression via induction of G2 arrest. Ovarian cancer cells with deficient p53 function have aberrant G1/S regulation and rely more heavily on G2/M regulation. Wee1 activates the G2/M checkpoint through phosphorylation of CDK1, a critical regulator for cells with deficient p53 function.

The serine/threonine kinase ATR functions as one of the cell’s master regulators of genotoxic stress. Some forms of DNA damage can result in tracts of single-stranded DNA. In addition, segments of single-stranded DNA may be formed at stalled replication forks when the activities of the replication helicase and DNA polymerase are uncoupled^[41]^. Replication protein A (RPA) is recruited to regions of single-stranded DNA (ssDNA). ATR then localizes to regions of single-stranded DNA (ssDNA) via its binding partner ATRIP (ATR interacting protein), a process dependent on RPA [Figure 1]^[42]^. Following ATR localization to RPA, multiple activator proteins, including TopBP1 and ETAA1, are required for ATR activation^[41]^. When active, ATR phosphorylates its immediate downstream effector CHK1. Moreover, ATR plays a key role in preventing replication fork collapse by limiting CDK (cyclin-dependent kinase) signaling, which restrains replication fork firing^[43]^. ATR also directly targets helicases, preventing unstable replication fork configurations, and ATR regulates deoxyribonucleotide availability in response to DNA damage. It has been proposed that replications forks may be more prone to collapse without these interventions^[41]^.

The high burden of replication stress in cancer cells suggests that malignant cells may be especially sensitive to ATR inhibition. To this end, ATR inhibitors have proven effective in slowing the proliferation of *BRCA2*-mutant HGSOC cell lines and tumors^[44]^, with synergistic effects following co-treatment with platinum-based chemotherapy or PARP inhibition. Using organoid models of HGSOC, recent studies have leveraged the ability to monitor homologous recombination defects to predict sensitivity to ATR inhibition^[45,46]^.

ATR inhibition has been studied in ovarian cancer as a mechanism for overcoming PARPi resistance^[47]^. Supporting this hypothesis, PARPi-resistant, BRCA1-null cells have been shown to increase dependence on ATR^[48]^. Two trials investigating combined ATR and PARP inhibition among patients with PARPi-resistant recurrent ovarian cancer are ongoing. CAPRI is a phase II clinical trial of olaparib in combination with the ATR inhibitor ceralasertib (AZD6738) in patients with recurrent ovarian cancer; data on the platinum-resistant ovarian cancer cohort of this trial has been published^[49]^. Among the 12 PARPi-naïve patients who were evaluated, the best response was stable disease in nine patients and progressive disease in three [Table 1]. This combination was well tolerated in the trial with a side effect profile similar to PARP inhibition alone. NCT04149145 is a phase I trial of niraparib in combination with the ATR inhibitor M4344 among patients with PARPi-resistant, recurrent ovarian cancer and was anticipated to begin enrollment in December 2022 (NCT04149145).

**Table 1:** Summary of key trials targeting ATR, CHK1, and WEE1 for the treatment of recurrent ovarian cancer

**Target**	**Inhibitor**	**Trial Identifier**	**Trial Overview**	**Key Findings**	**Reference**
ATR	Ceralasertib	NCT03462342	Phase II study of olaparib in combination with ceralasertib in platinum-resistant ovarian cancer	Among the 12 PARPi-naïve patients who were evaluable for response, the best response was stable disease in 9 patients.	[49]
CHK1	Prexasertib	NCT02203513	Phase II trial of prexasertib for recurrent ovarian cancer in patients without germline *BRCA*	Partial response rate of 29% (95% CI 13%-49%)	[64]
CHK1	Prexasertib	NCT02124148	Phase Ib study of prexasertib in combination with multiple chemotherapeutic agents in advanced cancer	No ovarian cancer-specific response data availableObjective response rate of 12.7% in combination with cisplatin armHematologic toxicities were dose-limiting.	[65]
CHK1	Prexasertib	NCT03057145	Phase I study of prexasertib in combination with the PARPi olaparib in HGSOC and other solid tumors	Identified a schedule with acceptable tolerabilityPartial response rate of 22% in PARPi-resistant HGSOC	[66]
WEE1	Adavosertib	NCT01164995	Phase II study of adavosertib plus carboplatin in patients with *TP53*-mutant advanced ovarian cancer, platinum-resistant or -refractory	ORR of 43%Median PFS of 5.3 monthsMedian OS of 12.6 months	[77]
WEE1	Adavosertib	NCT01357161	Phase II study of adavosertib versus placebo plus carboplatin and paclitaxel in patients with advanced, *TP53*-mutated platinum-sensitive ovarian cancer	Modest PFS prolongation in the combination arm: PFS (7.9 months *vs*. 7.3 months), HR 0.63 (0.38-1.06), *P* = 0.080)No significant change in ORR (74.6% *vs*. 69.4%, *P* = 0.52) or OS (HR 1.0 (0.53-1.88), *P* = 0.898)Increased adverse events in experimental arm	[78]
WEE1	Adavosertib	NCT02101775	Phase II randomized trial of adavosertib versus placebo with gemcitabine chemotherapy in patients with platinum-resistant or refractory HGSOC	Improved PFS in experimental arm (4.6 months *vs*. 3.0 months, HR 0.55 (0.35-0.90), *P* = 0.015)Improved OS in experimental arm (11.4 months *vs*. 7.2 months, HR 0.56 (0.35-0.91), *P* = 0.017) Improved partial response rate in experimental arm (23% *vs*. 6%, *P* = 0.038)Increased adverse events in experimental arm	[79]
WEE1	Adavosertib	NCT02272790	Adavosertib with chemotherapy in patients with primary platinum-resistant ovarian, fallopian tube, or peritoneal cancer: an open-label, four-arm, phase II study	The best ORR was 66.7%, with a disease control rate of 100%. Toxicity was notable. 100% of patients had grade 3 or higher adverse events.	[80]

ATR: Ataxia telangiectasia and Rad3 related; BRCA: breast cancer gene; CHK1: checkpoint kinase 1; CI: confidence interval; HGSOC: high-grade serous ovarian cancer; HR: hazard ratio; PARPi: poly (ADP-ribose) polymerase inhibitor; PFS: progression-free survival; ORR: overall response rate; OS: overall survival ratio.

## CHK1

The serine/threonine kinase CHK1 plays an integral role in the cellular response to genotoxic stress, functioning as the principal effector for ATR. CHK1 has been described in multiple organisms to regulate cell cycle transition during basal states^[50]^ and in response to DNA damage^[51-53]^. In addition, CHK1 regulates other proteins involved in DNA replication, including PCNA (proliferating cell nuclear antigen)^[54]^ and Pol-α (holding cells at the G2 phase) [Figure 1]^[55]^. Thus, activated CHK1 slows down DNA replication, allowing the cell to begin DNA repair, which is further enforced by CHK1 through its phosphorylation of Rad51, which is important in regulating HRR^[56]^. Given its role in cell cycle progression, CHK1 is heavily regulated, with phosphorylated CHK1 undergoing a transition from the nucleus to the cytoplasm and proteasome-mediated degradation^[57]^.

The underlying hypothesis for targeting CHK1 stipulates that blocking CHK1 activity will promote cell cycle progression in the presence of DNA damage, resulting in an accumulation of double-stranded breaks in rapidly dividing cancer cells. This accumulated damage will ultimately lead to collapse of genomic integrity and cell death. HGSOC is a particularly good candidate for CHK1 inhibition because p53-deficient cells will be preferentially dependent on the G2-M checkpoint which may render them further susceptible to CHK1 inhibition^[37,38]^.

To date, a majority of CHK1 inhibitors are ATP (adenosine triphosphate)-competitive and have been designed to have good selectivity over CHK1’s highly homologous cousin CHK2 (checkpoint kinase 2)^[58]^. Multiple first-generation CHK1 inhibitors did not advance through early phase clinical trials due to unfavorable pharmacokinetic and pharmacodynamic profiles, as well as excess toxicity. However, a second-generation CHK1 inhibitor (CHK1i), prexasertib (LY2606368), has emerged as a prioritized compound for further development^[59]^. Prexasertib is a selective small molecule inhibitor of CHK1 and CHK2^[60]^. Prexasertib^[61]^ showed efficacy as a monotherapy in HGSOC patient-derived PDX models which were *BRCA1*-mutant or resistant to olaparib, with further synergy observed between a PARPi and CHK1i^[62]^.

Prexasertib is currently advancing through clinical trials. It demonstrated favorable tolerability in a phase I clinical trial^[63]^. A phase II trial of prexasertib monotherapy for the treatment of recurrent HGSOC in patients without germline *BRCA* mutations demonstrated a partial response rate of 29% (95%CI: 13-49)^[64]^, with an encouraging response rate in the platinum-resistant or platinum-refractory patient subgroup of 32%. Common treatment-emergent adverse events included neutropenia, leukopenia, thrombocytopenia, and anemia [Table 1]^[64]^. These results are notable in this difficult-to-treat patient population and warrant further clinical investigation.

As described above, CHK1 is hypothesized to synergize with other agents that either induce DNA damage or inhibit its repair, such as chemotherapeutics and PARP inhibitors. A phase Ib study of prexasertib in combination with multiple chemotherapeutic agents (cisplatin, cetuximab, pemetrexed, or 5-fluorouracil) in patients with advanced or metastatic cancers reported an objective response rate of 12.7% in the cisplatin arm, with frequent and dose-limiting hematologic toxicities^[65]^. This approach has not been studied specifically in the platinum-resistant HGSOC population. A phase I study of prexasertib in combination with the PARPi olaparib in HGSOC and other solid tumors identified a schedule with acceptable tolerability and demonstrated preliminary activity in patients with *BRCA* mutations who had experienced prior progression on a PARPi [Table 1]^[66]^.

## WEE1

WEE1 is a tyrosine kinase that activates the G2/M cellular checkpoint through phosphorylation and subsequent inhibition of CDK1 (cyclin-dependent kinase 1) and CDK2 (cyclin-dependent kinase 2), which regulate cell cycle progression in the presence of damaged DNA [Figure 1]^[67,68]^. While normal cells repair damaged DNA during G1 arrest, malignant cells with deficient p53 and defects in the G1 checkpoint depend more on a functional G2–M checkpoint for DNA repair. Thus, inhibition of WEE1 can increase genomic instability and replication stress leading to mitotic catastrophe in cells with an overreliance on the G2/M checkpoint. Moreover, WEE1 levels are elevated in ovarian cancer^[69]^. Inhibition of WEE1 has also been shown to force cells arrested in S-phase into mitosis^[70]^. As such, WEE1 inhibitors have been hypothesized to synergize with DNA-damaging agents. This effect may be particularly relevant for p53-deficient cells, which are more dependent on the intra-S-phase checkpoint following DNA damage^[70]^.

In 2009, Hirai *et al.* reported on the development of adavosertib (MK-1775, AZD1775), a potent and selective small molecule inhibitor of WEE1^[71]^. Treatment with adavosertib sensitized cells to the antitumor effects of chemotherapy, with the largest effects noted, as would be expected, in p53 deficient cell lines^[71]^. Additional preclinical studies confirmed the sensitivity of *TP53-*mutant cells to the combined effects of chemotherapy plus adavosertib^[72-74]^ in non-ovarian cancer models, thus forming the basis for early phase I studies. Adavosertib was shown to negatively impact cell viability in both *in vitro* and *in vivo* models of ovarian cancer^[75]^.

In clinical trials, adavosertib has been studied as a monotherapy or in combination with chemotherapy in advanced solid tumors^[76]^. Encouraging responses were noted in tumors with mutant *TP53*, leading to a follow-up phase II study in HGSOC, which harbors high rates of inactivating *TP53* mutations^[33]^. A phase II study of adavosertib plus carboplatin in patients with *TP53*-mutant advanced ovarian cancer, either resistant or refractory to first-line platinum-based chemotherapy, demonstrated an encouraging overall response rate (ORR) of 43% [Table 1]^[77]^. Significant toxicities were noted, including frequent grade 3 or 4 thrombocytopenia and neutropenia.

A double-blind, randomized, phase II trial of the combination of adavosertib versus placebo plus standard platinum-based chemotherapy in patients with advanced, *TP53*-mutated platinum-sensitive ovarian cancer^[78]^ failed to demonstrate a significant difference in objective response rate (74.6% *vs.* 69.4%, *P* = 0.52). A randomized phase II trial of adavosertib versus placebo with gemcitabine chemotherapy in patients with platinum-resistant or platinum-refractory HGSOC showed a modest improvement in PFS (4.6 months *vs*. 3.0 months, HR 0.55 (0.35-0.90), *P* = 0.015) but with increased toxicity in the combination arm [Table 1]^[79]^.

Most recently, Moore *et al.* reported on the results of an open-label four-arm phase II trial of adavosertib in combination with chemotherapy in patients with primary platinum-resistant ovarian, fallopian tube or primary peritoneal cancer^[80]^. The best ORR was 66.7% (disease control rate 100%), but toxicity was considerable in this arm, with 100% of patients experiencing grade 3 or higher adverse events [Table 1]. In addition to these studies demonstrating a promising role of WEE1 inhibitors in combination with chemotherapy, a number of trials [NCT02576444 (OLAPCO), NCT03579316] have been planned or are ongoing to investigate adavosertib in combination with PARP inhibitors due to the role of WEE1 in stabilization of replication forks^[67]^. This combination has shown synergy in preclinical models; however, toxicity concerns persist^[81]^.

## MORE SELECTIVE AND POTENT EXPLOITATION OF HRD

The first generation of PARP inhibitors (olaparib, niraparib, rucaparib, and talazoparib) inhibit both PARP1 and PARP2. It has been proposed that PARP1 inhibition is required to induce DNA damage^[82]^. Moreover, inhibition of PARP2 has been linked to suppression of erythropoiesis^[83]^, and has been suggested to be a driver of the hematologic toxicity associated with the currently approved PARP inhibitors^[84]^. The first next-generation PARPi to enter clinical trials AZD5305 is a highly potent and selective PARP1 inhibitor^[84]^. AZD5305 is being studied in the ongoing PETRA study (NCT04644068), a phase I/II study of AZD5305 in patients with tumors harboring mutations in key DDR genes. Preliminary data demonstrated a favorable toxicity profile and encouraging clinical activity; publication of final results is highly anticipated. A PARP inhibitor with a superior therapeutic index may confer improved clinical activity as monotherapy. Moreover, an improved hematologic toxicity profile may allow for more effective and better tolerated combinations with other DNA damage repair inhibitors. Further clinical data are needed to evaluate these hypotheses.

## BIOMARKERS PREDICTIVE OF RESPONSE

Konstantinopoulos *et al.* reported a post-hoc retrospective study investigating patients with HGSOC receiving gemcitabine with berzosertib (an ATR inhibitor) *vs*. placebo. They observed that the benefit from the addition of berzosertib was only seen in patients with a platinum-free interval of 3 months or less. They proposed that tumors with low replication stress (defined as the absence of CCNE1 amplification, RB1 two-copy loss, CDKN2A two-copy loss, KRAS amplification, NF1 mutations, ERBB2 amplification, MYC amplification, and MYCL1 amplification) preferentially benefit from the addition of ATR inhibition to chemotherapy^[85]^. Further validation of this proposed biomarker approach is still needed.

*CCNE1*(cyclin E1) amplification may be another potential predictive biomarker. CCNE1 forms a complex with CDK2, and when activated, this complex is an important regulator of the initiation of DNA replication. *CCNE1* amplification has been associated with an acceleration of progression through the G1/S restriction point in the cell cycle, ultimately leading to an increase in mutations in genes that control cell survival and proliferation^[86]^. *CCNE1* amplification has been proposed as a biomarker of intrinsic resistance to platinum-based chemotherapy in ovarian cancer^[86]^. More recently, *CCNE1* amplification has been proposed as a potential biomarker indicative of response to combined WEE1 inhibition and ATR inhibition in preclinical ovarian cancer and endometrial cancer models. It has been proposed that *CCNE1-*overexpressing cells are preferentially dependent on the G2/M checkpoint. Both WEE1 and ATR are key regulators of the G2/M checkpoint, and as such, their inhibition may exploit a vulnerability in *CCNE1-*overexpressing cells that chemotherapy does not^[87]^. These results will require clinical validation, and perhaps more importantly, a combined WEE1-ATR inhibition strategy must demonstrate acceptable tolerability.

## CONCLUSION

In this review, we have explored several approaches to overcome platinum-based chemotherapy resistance and PARPi resistance, focusing on the role of DNA damage repair inhibitors. Several DNA damage repair inhibitors have demonstrated activity, but efficacy in the platinum-resistant setting has been relatively modest. Moreover, considerable hematologic toxicity has been a recurring limitation of this approach. Work is being done to identify patients most likely to benefit from this treatment approach, and the possibility of combination treatment strategies with a next-generation PARPi holds some promise. Based on the available data, it is almost certain that additional treatment strategies will be needed to overcome resistance to first-line treatment more effectively.

Combination treatment strategies might be most effective if employed as part of first-line treatment to address intrinsic rather than acquired resistance to platinum chemotherapy and PARP inhibition. Moreover, we emphasize that the identified resistance mechanisms to both platinum-based chemotherapy and PARP inhibition rely on much more than the roles of ATR, CHK1 and WEE1 in the DNA damage response. For example, a recent study identifies a druggable nucleus-to-mitochondria reactive oxygen species (ROS) sensing pathway, which appears to mediate resistance to platinum-based chemotherapy in ovarian cancers^[88]^. The identification of a novel mechanism of resistance holds promise, but further research to identify its best clinical application is needed. Multimodal combination approaches incorporating newer classes of medications, including antibody-drug conjugates and novel immunotherapy constructs, hold promise as well.

## DECLARATIONS

### Copyright

© The Author(s) 2023.
